# Development and validation of a guideline on sexual and reproductive health services for polycystic ovary syndrome in Iran: a mixed-methods study protocol

**DOI:** 10.1186/s12961-021-00793-z

**Published:** 2021-12-11

**Authors:** Mehri Kalhor, Eesa Mohammadi, Shadab Shahali, Leila Amini, Lida Moghaddam-Banaem

**Affiliations:** 1 Department of Reproductive Health and Midwifery, Faculty of Medical Sciences, Tarbiat Modares University, Jalal Al-e-Ahmad Highway, 14115-111 Tehran, Iran; 2 Department of Nursing, Faculty of Medical Sciences, Tarbiat Modares University, Tehran, Iran; 3 Nursing Care Research Center (NCRC) and School of Nursing and Midwifery, Iran University of Medical Sciences, Tehran, Iran

**Keywords:** Sexual and reproductive health, Polycystic ovary syndrome, Clinical practice guideline, Mixed-methods study, Study protocol

## Abstract

**Background:**

Sexual and reproductive health (SRH) is an important aspect of women's health. Polycystic ovary syndrome is a common disease among women and has long-term negative effects on women’s health. Evidence shows that polycystic ovary syndrome has different impacts on SRH needs among women. The aim of this study is to design and validate an SRH services guideline for healthcare providers in treating women with polycystic ovary syndrome.

**Methods:**

The guideline will be developed and validated using an exploratory sequential mixed-methods approach in three phases based on the National Institute for Health and Care Excellence (NICE) model: (1) scoping phase (describing the SRH needs of women with polycystic ovary syndrome from the results of both review and qualitative studies); (2) development phase (developing a primary guideline for SRH services); (3) validation phase (validation of the guideline will be performed by a panel of experts and stakeholders using the AGREE [Appraisal of Guidelines for Research and Evaluation] tool).

**Discussion:**

A specific and practical guideline on the SRH of Iranian women with polycystic ovary syndrome will be developed, which will be compatible with their specific needs and culture, considering the limited resources available. It will help service providers identify and address the specific needs of women with polycystic ovary syndrome.

## Background

Sexual and Reproductive Health (SRH) is one of the most important aspects of every woman’s life. The concept of reproductive health is an accepted part of public health worldwide [1, 2]. It covers a wide range of health services throughout an individual’s lifetime and aims to help individuals and families improve their health status [3, 4]. SRH care for women is not yet available in many countries, and according to published evidence on the burden of diseases, 22% of mortality in women of reproductive age is due to neglect of reproductive health issues [5, 6]. An important reproductive health condition that affects various aspects of women’s health is Polycystic Ovary Syndrome (PCOS), which is the most common endocrine disorder in women and also the most common cause of infertility because of anovulation. This syndrome is a combination of hyperandrogenism, chronic anovulation and polycystic ovaries. It is usually associated with insulin resistance and obesity and has a high prevalence (varying between 2.2 and 26% in different countries) [7, 8]. Evidence has shown that quality of life in women with PCOS is lower than that in other women [9].

In recent decades, the focus on quality of health services has increased. However, despite the significant success of health systems in promoting public health, there is still a considerable gap in achieving desirable results in the SRH domain [10]. Therefore, taking a comprehensive approach to SRH enables service providers to identify needs and to address them [11].

In countries with clinical standard practice guidelines, effective changes have been made in public health promotion [12]. Currently, there are several treatment guidelines for PCOS and also some for hirsutism [13–17]. Published guidelines emphasize therapeutic and clinical aspects of infertility in patients, and there are no specific clinical guidelines for the SRH needs of patients with PCOS [18–20]. Although several studies have been conducted on the issues of family planning [21], sexual health [22, 23], childbearing [24] and quality of life [25] of patients with PCOS, a comprehensive search of the literature performed by the authors thus far revealed no specific guidelines for meeting the overall SRH needs of women with PCOS.

Women with PCOS generally receive the usual SRH services; however, the needs and concerns of these women vary greatly from those of healthy women due to disease complications. Therefore, it is imperative that reproductive health professionals address the specific SRH needs of these women.

Since the prevalence of PCOS in Iranian women is 19.5% based on Rotterdam criteria [26], and given that the resources of the health system are limited, identifying and meeting the SRH needs of this rather large population of women can help improve their health and reduce the consequences of the disease during their reproductive period. Therefore, the development of an SRH guideline for these women is needed.

Since the purpose of this study is to develop a national guideline, key stakeholders from the Iranian Ministry of Health and Medical Education and relevant experts from all over the country will be present as members of the scientific committee in the study.

Although the ultimate goal of this study is to develop a guideline to be used at the national level in the Iranian health system, because of the common manifestations and complications of the disease in women in all areas of the world, it can also be used globally.

### Aim

The aim of this study is to develop and validate a comprehensive guideline for SRH services in women with PCOS in Iran, considering local and cultural needs.

## Methods

### Study design

**Fig. 1:**
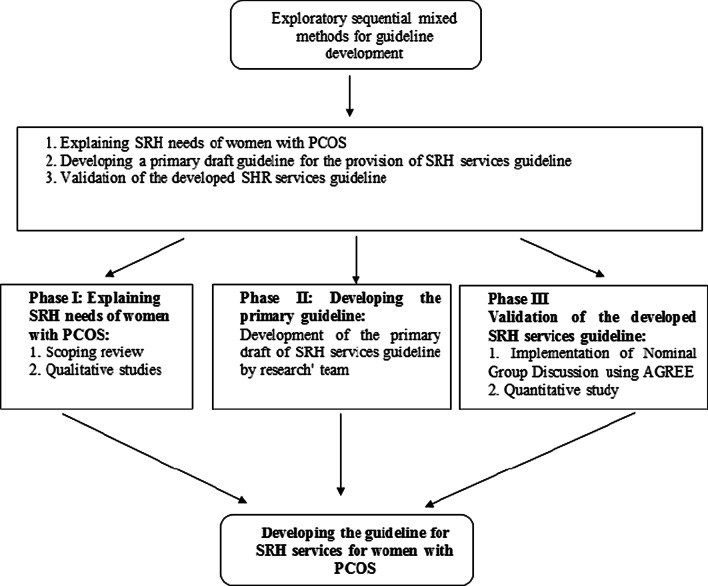
Study design

This study will use an exploratory sequential mixed-methods approach starting with qualitative data collection and analysis, followed by quantitative data collection and analysis, and finally combining the previous steps. This study will use the National Institute for Health and Care Excellence (NICE) model template. This model comprises four stages: (1) scoping, (2) development, (3) validation, and (4) publication and dissemination [27]. The three phases of the current research will be consistent with the first three stages of the NICE model, as described below. The design of the study is illustrated in Fig. 1.

#### Phase I: scoping phase

In phase I, the scope of the guideline will be determined. Therefore, the SRH needs of women with PCOS must be identified. In this phase, we will collect data to explore SRH needs in women with PCOS based on two studies: (1) a review study and (2) a qualitative study. During this phase, we will identify which needs and services are available in Iran, and whether women with PCOS have any difficulties accessing these services.

#### Phase I: scoping phase

##### A—review study

In this stage, a systematic review will be conducted to determine the SRH needs of women with PCOS. The study will be performed based on English or Persian peer-reviewed and grey literature sources from 2000 to 2021. This step will address the lack of a comprehensive study to examine the SRH needs of women with PCOS reported in the literature.

### Search engines and time period

Documents will be selected for the period from 1 January 2000 until the end of 2021. The following databases will be reviewed: Elsevier, Guideline Central, Johns Hopkins Medicine, Oxford Journals, BMJ, Springer, Ovid, MEDLINE, Science Direct, ProQuest, Scopus, EBSCOhost, Cochrane Library, PubMed and Google Scholar. National medical databases including SID, Magiran, IranDoc and IranMedex will also be reviewed.

### Search strategy

For a comprehensive exploration, the search terms will comprise several keywords, including various forms of PCOS and SRH. The following keywords will be used: “sexual and reproductive health needs”; “women”; “polycystic ovary syndrome”; “health needs”; “health guidelines”; “health protocols”; “women’s needs”; “women’s health needs”; “reproductive health needs”. The research team will combine terms within each category by “OR” and between categories by “AND”. Medical Subject Headings (MeSH) terms will be used only for searching in PubMed, and the other terms will be used in all databases. The search strategy is summarized in Table 1.

**Table 1:** The literature search strategy

Category	Term
Sexual and reproductive health	“Sexual health” [MeSH] “Sexuality” [MeSH] “Reproductive health” [MeSH] “Reproductive health services” [MeSH] “Fertility” [MeSH] “Fertility preservation” [MeSH] “Contraception” [MeSH] “Infertility” [MeSH] “Reproduction” [MeSH] “Sexual health” “Reproductive health” [All Fields] Sexuality “Sexual function” “Sexual health services” “Sexual dysfunction” “Reproductive health” “Reproductive health services” Fertility “Fertility preservation”
PCOS	“Polycystic ovary syndrome” [MeSH] “Polycystic” [all fields] “Ovary” [all fields] “Polycystic ovary syndrome” [all fields]

### Inclusion and exclusion criteria

Articles eligible for inclusion in the study will be English or Persian peer-reviewed and grey literature sources since 1994 about PCOS patients’ SRH needs. Studies about other diseases or groups will be excluded.

### Reduction of bias

The articles will be independently studied by two researchers (MK and one other member of the research team) for quality appraisal and relevance assessment; the related papers will be identified. In the case of one or more disagreements, the article will be reviewed by another researcher; a double-check process of validation will be performed to minimize the risk of bias. Also, the risk of bias in the included studies will be assessed by searching on multiple different websites and platforms.

### Quality assessment

MK and one other member of the research team will independently screen titles and abstracts of selected articles to ensure the relevance of title, abstract and keywords with the goals of this study. Subsequently, full-text review of selected articles will be conducted. Any disagreement will be resolved by a third reviewer. The quality of selected articles will be assessed using the Mixed Methods Appraisal Tool (MMAT) [28] and AMSTAR (A Measurement Tool to Assess Systematic Reviews) checklist [29].

### Data extraction and analysis

A datasheet with information including title, year, method, participants, author(s) or organization name, results and relation to SRH needs will be prepared for all the selected articles. Then data will be analysed using a thematic analysis method. This method develops new themes from the categories and primary codes derived from articles [30]. The analysis in the present paper will focus on identifying and categorizing SRH data for women with PCOS. In this way, the research team will extract the primary coding from the results and content of the selected articles and at the same time will place similar codes into the special categories. Research team members will also discuss their ideas and interpretations throughout the process. Two reviewers from the research team will independently extract data from eligible studies and convert them to primary codes and then compare the results. Finally, all primary codes will be assessed by the research team and, if all members agree, the primary codes will be categorized. Finally, themes will be extracted from the primary codes and categories.

#### Phase I: scoping phase B—qualitative study

The experiences of women with PCOS and service providers regarding SRH needs will be assessed through directed content analysis. This method was introduced by Hsieh and Shannon in 2005 [31]. In directed content analysis, researchers use existing theories or previous studies to identify key variables or concepts for the classification of primary codes. The main themes of the study are derived from existing theories or previous studies. If extracted primary codes cannot be placed in any of the main themes, new themes are created from the primary codes. The directed content analysis will be performed through in-depth interviews. Since this method requires a predefined theory, the theory used in this study is an existing questionnaire to explain SRH needs in women, the Sexual and Reproductive Health Needs Assessment. This questionnaire was designed by the United Nations Population Fund (UNFPA) in Zimbabwe in 2008 and has six main domains: (1) safe motherhood, morbidity profile and hygiene practices, (2) family planning, (3) sexual history and practices, (4) sexually transmitted infections, (5) HIV and AIDS, and (6) sexual and gender-based violence [32]. A semi-structured questionnaire will be used to conduct the interviews based on these six domains. Questionnaire structures and questions for women with PCOS and service providers are summarized in Table 2.

**Table 2:** Questionnaire structure and questions for women with PCOS and service providers

Themes derived from UNFPA questionnaire	Questions	
	Women with PCOS	Service providers
Safe motherhood, morbidity profile and hygiene practices	What motherhood (pre-, during- and postpartum care) problems have you experienced after PCOS diagnosis?	What are the needs of patients for safe motherhood?
Family planning	What concerns you in family planning?	What are family planning needs and priorities in women with PCOS?
Sexual history and practices	How has the disease affected your sexual relations?	What are the needs of patients in this field and what services do they receive?
Sexually transmitted infections	Have you ever experienced a sexually transmitted infection and what did you do about it?	Can PCOS affect sexually transmitted infections? How has your experience been with your patients?
HIV and AIDS	Do you know about HIV and AIDS? Have you experienced this disease?	Can PCOS affect HIV and Aids? If so, how?
Sexual and gender-based violence	What experiences of violence do you have?	Does PCOS affect violence against women, and if so, how?

### Data sources and sampling

Participants in the qualitative study will be women with PCOS, specialists and service providers for patients with PCOS (policy-makers and decision-makers involved in PCOS at the Ministry of Health and Medical Education, endocrinologists, gynaecologists, psychiatrists, nutritionists, dermatologists, reproductive health specialists and midwives). The purposive sampling method and a maximum variance approach will be used to select participants. Exploratory and probing questions will be asked after asking about each domain of the questionnaire and receiving the answer. Finally, open-ended questions will be asked to identify more specific needs of patients—for example: Is there a problem or need that I have not asked you about and you have not explained? Please explain. At the beginning of each interview, the research objectives and the right of individuals to participate in the study or to refuse at any time will be explained to participants, informed consent will be obtained from them and they will be assured about the confidentiality of their information.

### Rigour

The four criteria described by Lincoln and Guba will be used to ensure truthfulness of the findings [33]. The research team will review the primary codes during every interview. Also, these codes will be queried again from participants for validation. Purposive selection of participants enables the transferability of findings with maximum variance and their description. In order to provide credibility, the method of permanent and ongoing engagement with the subject of research will be used.

### Analysis

Data analysis will be performed using the Hsieh and Shannon method [31]. After each interview, the investigator will listen carefully to the interviews and will transcribe them. Then the interview transcripts will be read several times to gain a deep and accurate understanding of the interview, and texts will be broken into smaller semantic units. The primary codes will then be extracted from the semantic units. Primary codes will be placed in several subcategories according to a primary matrix through directed content analysis based on the UNFPA SRH needs questionnaire. This process will continue until all subcategories and categories have been placed in predefined categories. Whenever a primary code cannot be placed in an existing category or subcategory, a new category or subcategory will be created. Interviews will continue until no new data are obtained and data saturation is achieved.

#### Phase II: developing a guideline for provision of SRH care to Iranian women with PCOS

The second phase of the study will be the development of a preliminary guideline for SRH services in women with PCOS which conforms to the second stage of the NICE model, namely the development phase. First, the research team will use the PIPOH technique to develop a set of important questions [34]. This technique includes five items: population of interest (women with PCOS in Iran), interventions of interest (SRH services), target professionals (service providers at different levels), expected outcomes (access to appropriate SRH services for women with PCOS), and healthcare setting and context (all public and private health and medical centres providing services to women with PCOS including healthcare centres, medical offices, clinics and hospitals).

The main question of the study will be as follows: What type of health services should women with PCOS receive regarding prevention, screening, diagnosis and management of their SRH problems?

Then, over the course of several meetings, the research team members will debate the results of the first phase, including the review study and qualitative study, to pool their understandings and findings and agree on a conclusion. For this purpose, the findings related to each theme of the review and qualitative studies will be compiled in the form of a table. The results of both studies will then be reviewed by the research team, and the main recommendations will be derived from the themes. In cases where the results of the two studies do not match, the issue will be discussed by the research team, and their consensus will determine whether the issue remains or is omitted based on its importance and the recommendations of other guidelines.

The research team will also be exploring the literature on guidelines and policies related to SRH needs and services for women with PCOS. MK and one other member of the research team will independently conduct a comprehensive literature review to find all relevant guidelines, protocols and documents published in English or Persian from 1 January 2000 through 2021 in medical literature databases, clinical guideline databases, international PCOS-related websites and national medical databases. Terms will be combined within each category by “OR” and between categories by “AND”. MeSH terms will be used only for searching in PubMed, and the other terms will be used in all databases. The research team will screen available guidelines to ascertain whether they are up to date and to confirm the validity of their sources. Guidelines for which the quality assessment score obtained by the AGREE [Appraisal of Guidelines for Research and Evaluation] tools is greater than 40% will be chosen. Evaluating the recommendations derived from literature reviews will be assessed by the Grading of Recommendations Assessment, Development and Evaluation (GRADE) framework [35]. The level of evidence for each recommendation derived from literature reviews will be assessed using the evaluation GRADE system in Table 3. Data for selected guidelines will be summarized in a table including their title, source, date of last update, target audience and results.

Then, for each SRH theme, the research team will tabulate the results of the first and second phases and make recommendations. Next, the research team will select recommendations regarding each question of the study based on their level of evidence, benefits (e.g., improving quality of life, decreasing morbidity among women with PCOS), harms (e.g., greater costs, increased morbidity and number of referrals) and compatibility with local and cultural circumstances based on phase I (review and qualitative studies) results. The important outcomes will be selected based on the results of the review and qualitative studies in phase I and the review of the scientific evidence in phase II. The outcomes that have a significant impact on SRH domains will be prioritized.

Three techniques will be used for making recommendations: adoption (accepting existing recommendations from available guidelines and evidence as they are), adaptation (modifying existing recommendations from available guidelines and evidence to meet local and cultural needs) and de novo development of recommendations if required (Iranian women with PCOS require a specific health service according to the phase I results but there is no available recommendation in this regard in the literature based on the phase II results) [36]. The strength of the recommendations will be determined according to the criteria in Table 4 [37]. Then the research team will prepare a table for the developed recommendations including source, level of evidence, grade of recommendation, benefits and harms. This table, with the supporting scientific evidence, will be shared among the scientific committee group consisting of a gynaecologist, endocrinologist, psychiatrist, nutritionist, dermatologist, reproductive health specialist, midwife and sexologist. The scientific committee will submit their opinions about each recommendation (agree, disagree, agree with some modifications) in written format. Finally, the research team will modify the guideline based on the opinions of the scientific committee. The guideline will be developed at the national level, and Iranian women with PCOS will be the target population.

The users of the guideline will include all healthcare professionals providing services to women with PCOS at different levels (e.g., endocrinologists, gynaecologists, psychiatrists, nutritionists, dermatologists, obstetricians, infertility specialists, reproductive health specialists, psychologists, psychiatrists, counsellors and midwives). Therefore, in the scientific committee, key stakeholders from the Iranian Ministry of Health and Medical Education and experts related to PCOS will be present from all over the country.

**Table 3:** Level of evidence

Level	Definition
1: Strong	Randomized clinical trials or their meta-analysis
2: Intermediate	Non-randomized clinical trials and their meta-analysis Case–control studies or their meta-analysis Prospective cohort studies
3: Weak	Cross-sectional studies, observational studies, case series or case reports
4: No evidence	Insufficient evidence

**Table 4:** Grade of recommendation

Grade	Definition
A	All level 1 evidence shows that benefits are greater than harms
B	At least one piece of level 1 and level 2 evidence shows that benefits are greater than harms
C	There is no high-level evidence that benefits are greater than or equal to harm. Decisions should be made based on experts’ opinions
D	There is evidence that harms are greater than benefits

#### Phase III: validation of the developed guideline for provision of SRH services to Iranian women with PCOS

Validation of the guideline will be carried out in concordance with the third step of the NICE model, namely the validation step. This phase will be performed using the nominal group technique (NGT) [38]. This meeting will be held with the participants including the research team, scientific committee, patient representatives, service providers and stakeholders. Experts from the PCOS centres and department for clinical guideline standardization from the Iranian Ministry of Health and Medical Education will also attend this meeting. All participants will be informed in advance, by email and in writing, regarding the aim, scope and process of guideline development. At the beginning of the meeting, MK will present a summary of the guideline development process, the opinions of the scientific committee, agreements and disagreements. The participants will then discuss the recommendations considering everyone’s opinions including stakeholders. The executive considerations including acceptability, accessibility and utilization of the health services will also be discussed. After the nominal group meeting, the research team will modify the guideline based on the results of the meeting. The recommendations with more than 80% agreement will be included in the guideline. For recommendations with less than 80% disagreement, the guideline development core group will decide based on results of evidence.

Finally, the guideline will be evaluated using the AGREE instrument in a quantitative approach. AGREE contains 23 items in six domains: scope and purpose, stakeholder involvement, rigour of development, clarity of presentation, applicability and editorial independence. Each item is rated on a four-point scale from 1 (strongly disagree) to 4 (strongly agree). A score is calculated independently for each of the six domains and is not accrued into a single score. Domain scores are useful for evaluating guideline quality [39]. Nominal group members will evaluate the guideline overall and will be asked whether they would recommend it. There will also be a space under each item for further comments. The evaluation will be performed in two steps. First, members of the nominal group will evaluate the guideline using the AGREE instrument. Second, to evaluate the practicality of the guideline, we will circulate it among at least 60 service providers, asking them the last question in the AGREE instrument as to whether they would recommend the use of the guideline and whether they have any suggestions for improvement. In the end, all suggestions and recommendations will be collected in written format. Subsequently, the comments and suggestions will be assessed by the research team and will be integrated into the final version of the guideline. The developed guideline will be updated every 5 years according to new scientific evidence.

## Discussion

Having access to services for SRH needs is an essential right of every woman in the world, and no woman should be deprived of this right due to any illness or other causes [1, 2]. Unfortunately, SRH needs for women in specific patient groups such as patients with PCOS have not been thoroughly addressed up to now; studies have mostly focused on the quality of life and sexual function/ satisfaction of these patients [40–42]. Therefore, providing SRH services for women with PCOS is necessary.

Various countries have undertaken many different strategies to provide SRH services to women, including establishing reproductive health clinics, integrating SRH programmes with other health programmes, and formulating SRH guidelines and protocols [43]. In addition to addressing the needs expressed, effective changes have also been achieved in improving public health. Therefore, providing a guideline to meet the needs of individuals seems to be a necessary step in promoting health [12]. Because PCOS is a condition that is present throughout a woman’s life, with specific problems at each age, providing SRH services to these women can improve their overall health and quality of life [25]. In a healthcare setting, a guideline—also called a medical guideline—is a set of instructions that describe a process to be followed to investigate a particular set of findings in a patient or the method which should be followed to control a certain disease [44]. The guidelines can predict appropriate services according to the severity and outcomes of the disease, for better disease management. On the other hand, the financial burden of a disease can also be reduced by following an appropriate guideline [8]. Special health guidelines can help health policy-makers consider individual, social and economic outcomes of a chronic disease in health planning. They can also contribute to better diagnosis, prevention and management of chronic multidimensional diseases such as PCOS [8]. Since there is currently no special guideline for SRH services for women with PCOS, the present study can be an example of a pioneer study in this field.

## Data Availability

The developed guideline will be available from the corresponding author on request.

## Abbreviations

SRH: Sexual and reproductive health
PCOS: Polycystic ovary syndrome
MMAT: Mixed Methods Appraisal Tool
AMSTAR: A Measurement Tool to Assess Systematic Reviews
AGREE: Appraisal of Guidelines for Research and Evaluation
GRADE: Grading of Recommendations Assessment, Development and Evaluation
NGT: Nominal group technique
NICE: National Institute for Health and Care Excellence
PIPOH: Population [of interest], intervention, professionals [delivering intervention], outcome, health setting
